# Introduction of the transmissible mobile colistin resistance genes *mcr-3* and *mcr-9* to the USA via imported seafood

**DOI:** 10.1128/msphere.00253-25

**Published:** 2025-07-07

**Authors:** Jouman W. Hassan, Tongzhou Xu, Marwan Osman, Steven J. Schiff, David Mann, Xiangyu Deng, Jeffrey T. LeJeune, Issmat I. Kassem

**Affiliations:** 1 Center for Food Safety, Department of Food Science and Technology, University of Georgia, Griffin, Georgia, USA; 2 Department of Neurosurgery, Yale University School of Medicine 5755, New Haven, Connecticut, USA; 3 The Food and Agriculture Organization (FAO) 17107, Rome, Italy; University of Nebraska Medical Center College of Medicine, Omaha, Nebraska, USA

**Keywords:** colistin, mcr, antibiotic resistance, seafood, plasmids, mobile genetic elements

## Abstract

The emergence and global dissemination of the mobile colistin resistance genes (*mcr*) threaten the efficacy of colistin, a high-priority, critically important antibiotic that is used to treat complicated infections with multidrug-resistant Gram-negative bacteria in humans. The occurrence of *mcr* in the USA has been suggested to be relatively limited, particularly in bacteria associated with domestic foods and food animals. This is because colistin has neither been marketed nor approved for use in agriculture in the USA. However, *mcr*-carrying bacteria can occur on foods imported from countries where these genes might be relatively more prevalent. Yet, studies on *mcr* in vulnerable imported foods in the USA are lacking. To address this gap in knowledge, we assessed the role of imported seafood as a potential carrier of *mcr* genes to the USA. Imported seafood samples were aseptically collected from eight major retail stores across Georgia, USA. In-depth analyses revealed the occurrence of *mcr-9* in bacteria isolated from imported shrimp samples. The *mcr-9*-carrying bacteria were identified as *Serratia nevei*, a newly described species that belongs to the *Serratia marcescens* complex. The *mcr-9* in the *S. nevei* isolates was carried on IncHI2 plasmids that were transferable and conferred colistin resistance to naïve *Escherichia coli*. Further analysis identified a chromosomal *mcr-3.17* in *Aeromonas salmonicida* isolated from imported scallops. All the *mcr*-carrying isolates harbored other important antibiotic resistance genes. Taken together, our data showed that imported seafood, specifically shrimps, might be an overlooked source contributing to the introduction and spread of transmissible colistin resistance genes in the USA.

**IMPORTANCE:**

Colistin, an important antibiotic, is used to treat certain bacterial infections in humans that can be severe and/or life-threatening. However, these bacteria can acquire the mobile colistin resistance (*mcr*) genes and become resistant to this antibiotic. Plasmid-borne *mcr* can jump between bacterial species, spreading in bacteria across a variety of hosts and niches. Therefore, monitoring the spread of *mcr* is critical to maintain the efficacy of colistin. In the USA, the occurrence of *mcr* in domestically produced food is thought to be limited. In this study, we showed that *mcr* can be carried into the USA by bacteria on imported seafood. A specific gene, *mcr-9*, was located on a plasmid that could be transferred to other bacteria. Therefore, imported seafood can be an overlooked source of *mcr* in the USA. It is important to monitor and assess *mcr* in imported seafood to control the proliferation of colistin resistance in the USA.

## OBSERVATION

The effectiveness of colistin has been threatened by the emergence of the plasmid-borne mobile colistin resistance genes (*mcr*) across the globe (1–3). In the USA, colistin was neither marketed nor approved by the US Food and Drug Administration for use in food animals (4). Subsequently, *mcr* genes are believed to have a relatively low prevalence and restricted dissemination in the USA (5–8). However, recent studies have reported the occurrence of *mcr* in sewage and effluent water samples, indicating that these genes might be circulating in human communities and the environment in the USA (9–11). The latter suggests that *mcr* might be relatively more prevalent in the USA than previously thought and/or being introduced into the USA via yet-unexplored routes. Previously, we suggested that trading food might facilitate the transmission of *mcr* between countries, including the USA (12, 13). Seafood is of particular interest because the USA imports 65%–85% of its seafood from other countries, and more than half of the imports are from aquaculture operations (14, 15). Notably, (i) *mcr* has been reported in bacteria isolated from seafood in the majority of these exporting countries, and (ii) aquaculture has been hypothesized as a source of *mcr* (5). Furthermore, the increasing importance of seafood as a potential carrier of antibiotic-resistant bacteria spurred the National Antimicrobial Resistance Monitoring System (NARMS) to start monitoring retail seafood in the USA in 2020. However, NARMS has focused only on specific bacterial species so far (15). Taken together, we hypothesized that *mcr* might be introduced into the USA by bacteria on imported seafood.

Imported (*n* = 45) and domestic seafood samples (*n* = 18) were aseptically collected from eight major retail stores across Georgia, USA. All the samples were refrigerated in the stores at the time of collection, mainly in the mornings (between 9:00 and 10:30 a.m.) shortly after the seafood displays were stocked. The samples were transported to the laboratory in a cooler with ice packs and processed within 2 h of collection. Each sample (~25 g) was suspended in 100 mL buffered peptone water (Oxoid, UK) and homogenized for 1 min at 225 rpm in a stomacher. An aliquot (100 µL) from each suspension was spread onto RAPID’*E. coli* 2 agar (Bio-Rad, USA) plates supplemented with 4 µg/mL of colistin (Sigma-Aldrich, USA), which were incubated at 37°C under aerobic conditions (10, 16). Putative colistin-resistant isolates were observed in 20% (*n* = 9/45) and 16.6% (*n* = 3/18) of the imported and domestic seafood samples, respectively. Forty-eight colonies (four isolates per sample) were randomly selected and screened for *mcr* (*mcr-1* to *mcr-10*) using gene-specific PCR (11). *mcr*-positive isolates were further analyzed using short- and long-read whole-genome sequencing (WGS) to identify bacterial species, antimicrobial resistance genes (ARGs), virulence genes, and plasmid types as described previously (11). Additionally, snippy v.4.4.5 (https://github.com/tseemann/snippy) and snp-dists v.0.8.2 (https://github.com/tseemann/snp-dists) were used to generate core genome alignments and calculate pairwise single-nucleotide polymorphism (SNP) distances between selected strains. The antimicrobial resistance (AMR) phenotypes of the *mcr*-positive isolates were determined against 19 clinically and agriculturally important antibiotics, including colistin, using the Kirby-Bauer disk diffusion and the broth microdilution assays as described in the guidelines of the Clinical and Laboratory Standards Institute (CLSI) (17). The transmissibility of the *mcr*-carrying plasmids to naïve *Escherichia coli* DH5-alpha was evaluated using the heat shock assay (11). Colistin resistance of the transformants was determined using gene-specific PCR analysis and the broth microdilution assay. Plasmids were extracted from the transformants and typed using the PCR-based replicon typing (PBRT) assay (16, 18). Lastly, the persistence of *mcr* was evaluated in 12-day-old biofilms as described previously (11).

PCR analyses detected *mcr* in six isolates (12.5% of the tested isolates) that were retrieved from six imported seafood samples (13.4% of the imported samples). Specifically, one *mcr-3*-positive isolate was retrieved from scallops imported from China, while five *mcr-9*-positive isolates were found in shrimps imported from Indonesia and Thailand (Table 1). WGS analyses corroborated the PCR results and identified the isolates as *mcr-3.17*-positive *Aeromonas salmonicida* (*n* = 1) and *mcr-9*-positive *Serratia nevei* (*n* = 5). The isolates exhibited high resistance to colistin, with a minimum inhibitory concentration (MIC) > 640 µg/mL (Table 1), which aligned with the intrinsic and variable colistin resistance reported for *Serratia* spp. and *Aeromonas* spp., respectively. The *mcr-9*-positive *S. nevei* were multidrug-resistant, with four isolates exhibiting resistance to penicillin, ampicillin, amoxicillin/clavulanic acid, tetracycline, and erythromycin, while *S. nevei* SFG-36 was also resistant to streptomycin (Table 1). The *mcr-9*-positive *S. nevei* carried ARGs that encoded resistance to aminoglycosides [*aac(6')-Ic*], cephalosporins (*bla*_SRT-2_), and tetracyclines [*tet(41*)], while *sul(1)* (sulfonamides), *aadA2b* (aminoglycosides), *tet(C)* (tetracyclines), and *qacE* (quaternary ammonium compounds) were also detected in SFG-36 (Table 1). Similarly, the *mcr-3.17*-positive *A. salmonicida* was multidrug-resistant and harbored two additional ARGs, *cphA5* (encodes an Ambler class B metallo-β-lactamase with specific activity against carbapenems) and *bla*_OXA-956_ (encodes an OXA-type beta-lactamase) (Table 1). In all cases, the detected ARGs corroborated the AMR phenotypes of the *mcr*-positive isolates (Table 1).

**TABLE 1:** Antibiotic resistance profiles and whole-genome sequence analysis of *mcr-3*- and *mcr-9*-positive bacteria isolated from imported seafood samples in Georgia, USA

Retail store/location^*a*^	Sample type (country of origin)^*b*^	Sample ID code	Bacterial identity^*c*^	Colistin MIC (µg/mL)	Transformant MIC (µg/mL)	Phenotypic antibiotic resistance profile (ABR) by disk diffusion assay^*d*^	ABR genes detected by ResFinder v.4.5 (% Identity)^*e*^ MyDbFinder v.2.0 (% identity)^*f*^	Additional ABR genes detected by CARD-RGI v.3.2.0 (perfect/strict)^*g*^	Plasmids detected by PlasmidFinder v.2.1 (% identity)^*h*^	Virulence genes detected by VirulenceFinder v.2.0 (% identity)^*i*^	Human pathogen predictor by PathogenFinder v.1.1^*j*^	Long-read Oxford Nanopore Technologies GridION accession no.	*mcr-9-*positive contig accession no. (plasmid accession no.)
Grocery store A/Griffin, Georgia, USA	Bay scallop 100/200 (China)	SFG-4	*Aeromonas salmonicida*	>640	No transformants	R: PEN-AMP-AMC-DOR-MEM-ERY I: IPM S: FEP-CTX-CFM-GEN-KAN-STR-TET-CIP-NOR-SXT-CHL	***mcr-3.17*** (99.38%); *cphA5* (96.01%) *bla_OXA-956_* (98.87%)	*mcr-3.17*; *cphA5*	None detected	None detected	No (0.26)	JAROCS000000000	None detected
Grocery store B/McDonough, Georgia, USA	White shrimp 51/60 (Indonesia)	SFG-21	*Serratia nevei*	>640	32	R: PEN-AMP-AMC-TET-ERY S: FEP-CTX-CFM-DOR-IPM-MEM- GEN-KAN-STR-CIP-NOR-SXT-CHL	*aac(6')-Ic* (96.37%); *tet(41*) (98.81%); *bla*_SRT-2_ (96.37%) ***mcr-9*** (100%)	***mcr-9.1***/*aac(6')-Ic*; *bla*_SRT-2_; *tet(41);crp; E. coli* GlpT with mutation conferring resistance to fosfomycin; *E. coli* EF-Tu mutants conferring resistance to pulvomycin	IncFIB(K) (98.93%); IncFII(Yp) (99.57%); **IncHI2** (100%); IncHI2A (100%)	*terC* (100%)	Yes (0.79)	JAROCT000000000	GCA_964241815 (PP341404.1)
Grocery store B/McDonough, Georgia, USA	Cooked, peeled, and ready-to-eat shrimp 41/50 (Indonesia)	SFG-24	*Serratia nevei*	>640	16	R: PEN-AMP-AMC-TET-ERY S: FEP-CTX-CFM-DOR-IPM-MEM- GEN-KAN-STR-CIP-NOR-SXT-CHL	*aac(6')-Ic* (96.37%); *tet(41*) (98.81%); *bla*_SRT-2_ (96.37%) ***mcr-9*** (100%);	***mcr-9.1***/*aac(6')-Ic*; *bla*_SRT-2_; *tet(41);crp; E. coli* GlpT with mutation conferring resistance to fosfomycin; *E. coli* EF-Tu mutants conferring resistance to pulvomycin	IncFIB(K) (98.93%); IncFII(Yp) (99.75%); **IncHI2** (100%); IncHI2A (100%)	*terC* (100%)	Yes (0.8)	JAROCU000000000	GCA_964239105 (PP341405.1)
	Shrimp red 16/25 (Thailand)	SFG-37	*Serratia nevei*	>640	2	R: PEN-AMP-AMC- TET-ERY S: FEP-CTX-CFM-DOR-IPM-MEM- GEN-KAN-STR-CIP-NOR-SXT-CHL	*aac(6')-Ic* (96.37%); *tet(41*) (98.81%); *bla*_SRT-2_ (96.37%) ***mcr-9*** (100%)	***mcr-9.1***/*aac(6')-Ic*; *bla*_SRT-2_; *tet(41);crp; E. coli* GlpT with mutation conferring resistance to fosfomycin; *E. coli* EF-Tu mutants conferring resistance to pulvomycin	IncFIB(K) (98.93%); IncFII(Yp) (99.75%); **IncHI2** (100%); IncHI2A (100%)	*terC* (100%)	Yes (0.8)	JAROCX000000000	GCA_964240345 (PP341408)
Grocery store C/McDonough, Georgia, USA	Shrimp red 16/25 (Thailand)	SFG-35	*Serratia nevei*	>640	2	R: PEN-AMP-AMC-TET-ERY S: FEP-CTX-CFM-DOR-IPM-MEM- GEN-KAN-STR-CIP-NOR-SXT-CHL	*aac(6')-Ic* (96.37%); *tet(41*) (98.81%); *bla*_SRT-2_ (96.37%) ***mcr-9*** (100%)	***mcr-9.1***/*aac(6')-Ic*; *bla*_SRT-2_; *tet(41);crp; E. coli* GlpT with mutation conferring resistance to fosfomycin; *E. coli* EF-Tu mutants conferring resistance to pulvomycin	IncFIB(K) (98.93%); IncFII(Yp) (99.75%); **IncHI2** (100%); IncHI2A (100%)	*terC* (100%)	Yes (0.8)	JAROCV000000000	GCA_964240015 (PP341406.1)
Grocery store D/McDonough, Georgia, USA	Large white shrimp on a skewer (Indonesia)	SFG-36	*Serratia nevei*	>640	32	R: PEN-AMP-AMC-STR-TET-ERY S: FEP-CTX-CFM-DOR-IPM-MEM- GEN-KAN-CIP-NOR-SXT-CHL	*aac(6')-Ic* (96.37%)*; aadA2b* (99.87%); *tet(41*) (98.73%)*; tet(C)* (99.91%)*; sul1*(100%)*; bla*_SRT-2_ (96.83%); *qacE* (100%) ***mcr-9*** (100%)	***mcr-9.1****; tet(C); sul1; aadA2/aac(6')-Ic*; *bla*_SRT-2_; *crp; E. coli* GlpT with mutation conferring resistance to fosfomycin; *E. coli* EF-Tu mutants conferring resistance to pulvomycin	IncFIB(K) (98.93%); IncFII(Yp) (99.75%); **IncHI2** (100%); IncHI2A (100%)	*terC* (100%); *traT* (99.04%); *clpK1* (99.64%)	Yes (0.75)	JAROCW000000000	GCA_964240025 (PP341407.1)

^*a*^ Samples were collected from different grocery stores (represented in letters A, B, C, and D) in Georgia, USA.

^*b*^ Type of seafood sample collected and the country of origin.

^*c*^ The identity was determined using the ribosomal multilocus sequence typing scheme (https://pubmlst.org/).

^*d*^ Resistance (R), intermediate (I), and susceptibility (S) to antibiotics were determined using the disk diffusion assay according to the Clinical and Laboratory Standards Institute (CLSI, 2025) guidelines. PEN, penicillin; AMP, ampicillin; AMC, amoxicillin/clavulanic acid; FEP, cefepime; CTX, cefotaxime; CFM, cefixime; DOR, doripenem; IPM, imipenem; MEM, meropenem; GEN, gentamicin; KAN, kanamycin; STR, streptomycin; TET, tetracycline; CIP, ciprofloxacin; NOR, norfloxacin; SXT, trimethoprim/sulfamethoxazole; CHL, chloramphenicol; ERY, erythromycin.

^*e*^ Acquired ABR genes were detected by the ResFinder v.4.5 database (http://genepi.food.dtu.dk/resfinder) using default settings.

^*f*^ MyDbFinder v.2.0 (https://cge.food.dtu.dk/services/MyDbFinder/) was used to confirm the detection of *bla_OXA-956_* in SFG-4 using the National Database of Antibiotic Resistant Organisms, and *mcr-9* was further confirmed using the phosphoethanolamine lipid A transferase gene family database available at NCBI.

^*g*^ ABR genes detected by the Comprehensive Antibiotic Resistance Database (CARD) v3.2.0 antibiotic resistance ontology database (https://card.mcmaster.ca/analyze/rgi) using default setting.

^*h*^ Plasmids detected by PlasmidFinder v.2.1 (https://cge.food.dtu.dk/services/PlasmidFinder/) using default settings. Plasmids carrying *mcr-9* are highlighted in bold.

^*i*^ Virulence genes; *terC* (encodes tellurium ion resistance protein), *traT* (outer protein complement resistance), and *clpK1* (heat shock survival AAA family ATPase ClpK, thermal stress survival) were detected by VirulenceFinder v.2.0 (https://cge.food.dtu.dk/services/VirulenceFinder/) using default settings.

^*j*^ Probability of being a human pathogen by Pathogenfinder v.1.1 (https://cge.food.dtu.dk/services/PathogenFinder/) using default settings.

**Fig 1:**
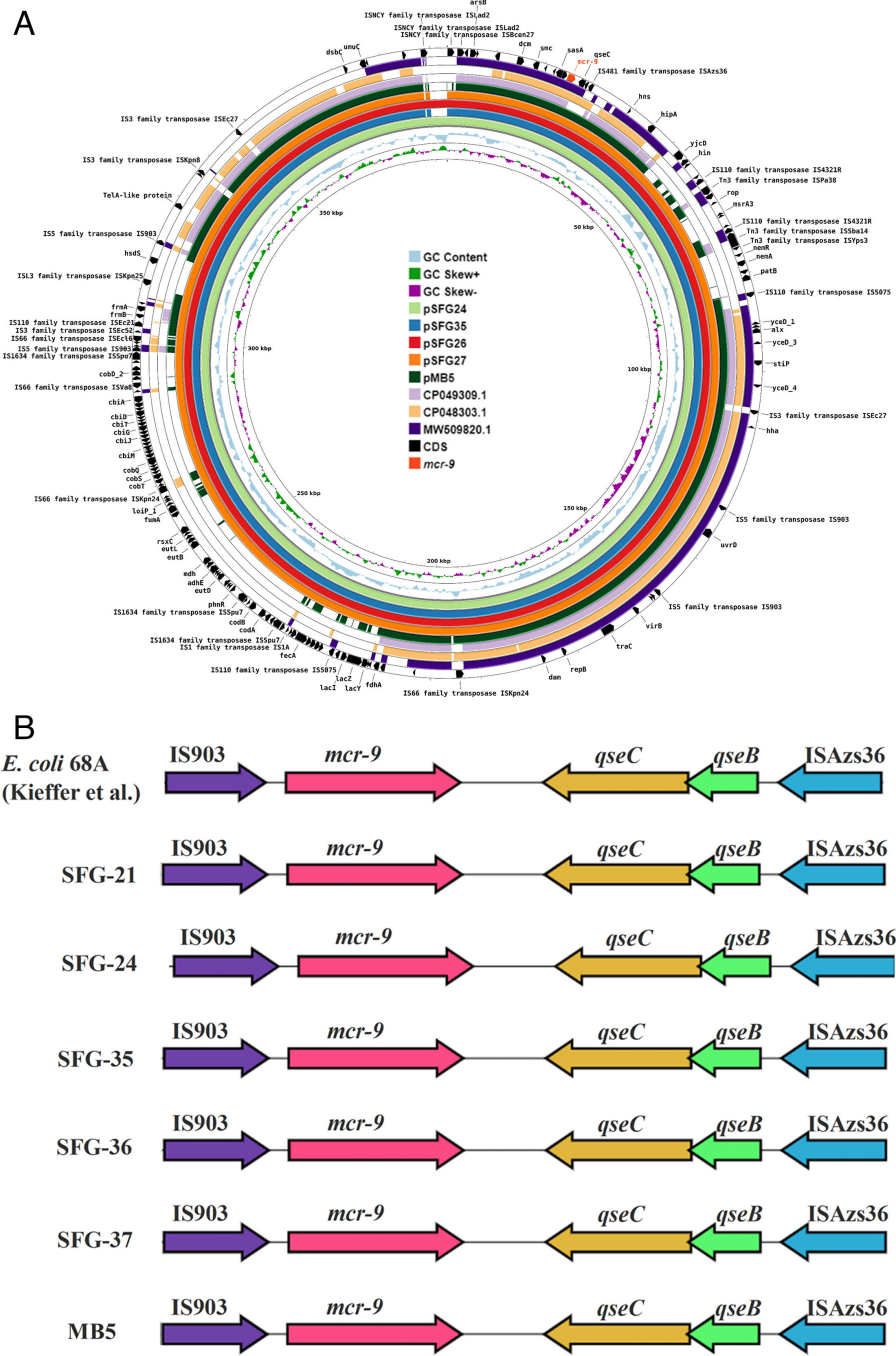
**(**A**)** Genetic characterization and comparative analysis of *mcr-9*-containing IncHI2 plasmids retrieved from *S. nevei* (pSFG-21, pSFG-24, pSFG-35, pSFG-36, pSFG-37) from imported seafood, with four *mcr-9*-containing IncHI2 plasmids retrieved from GenBank-NCBI. The plasmids belonged to *Salmonella* isolated from chicken breast (CP049309.1) and retail ground turkey (CP048303.1) from the USA, an *E. coli* from a clinical sample in Italy (MW509820.1), and *S. nevei* recovered from sewage influent in the USA (pMB5 [11]). GC skew (+) and GC skew (−) are depicted in green and purple, respectively. The plasmids were annotated by Prokka v.1.12, and the coding sequences (CD) are represented in black. (B) Alignment of the genetic context of *mcr-9-*positive contigs from our SFG-21 (JAROCT000000000), SFG-24 (JAROCU000000000), SFG-35 (JAROCV000000000), SFG-36 (JAROCW000000000), and SFG-37 (JAROCX000000000) isolates against *E. coli* 68 A (UILU00000000) and *S. nevei* recovered from sewage influent (pMB5 [JAZAPW000000000]). Alignments were generated using clinker (https://cagecat.bioinformatics.nl/) with a minimum alignment sequence of 80%.

Genomic analysis showed that *mcr-3* was localized on the chromosome of the *A. salmonicida* isolate, while the *mcr-9*-positive *Serratia* isolates harbored multiple plasmid types (Table 1). Subsequently, the *Serratia* isolates were investigated using GridION-ONT sequencing (11, 19), which revealed that *mcr-9* was carried on IncHI2 plasmids in all of the isolates (Fig. 1A). The latter was confirmed using the heat-shock assay, which resulted in the transfer of the *mcr-9*-carrying plasmids to *E. coli* DH5-alpha (Thermo Scientific, USA). The transformants were *mcr-9*-positive, and the *mcr-9*-carrying plasmids in the transformants were identified as IncHI2 using PBRT analysis. The colistin MIC of the transformants (ranging from 2 to 32 µg/mL) was higher than the *E. coli* epidemiological cutoff for colistin resistance (1.5 µg/mL) (20), indicating that *mcr-9* was plasmid-borne and expressed colistin resistance in otherwise naïve *E. coli*.

IncHI2 plasmids are widely distributed in *Enterobacteriaceae* and are associated with carrying and spreading multiple ARGs, including *mcr-9* (21). A comparative analysis of the *mcr-9*-carrying IncHI2 plasmids was performed to better understand the genetic context of *mcr-9* in these plasmids (Fig. 1A and B). We found that *mcr-9* was flanked downstream by the two-component regulator system *qseB/qseC* and an IS*481* family transposase, IS*Azs36*, and upstream by an IS*1* family transposase, IS*1X2,* in all the IncHI2 plasmids carried by the *S. nevei* isolates. The transposases likely facilitate the mobilization and transferability of *mcr-9* (20, 22). Furthermore, the presence of the *qseB*/*qseC* two-component regulatory system has been previously associated with the inducibility of *mcr-9* and expression of colistin resistance under specific environmental stress conditions and/or colistin selection pressure (20, 22).

The *mcr-9*-positive *S. nevei* were able to form biofilms (OD600 = 0.4–0.7). Furthermore, colonies (*n* = 500) randomly retrieved from 12-day-old biofilms were *mcr-9*-positive using PCR, indicating that the *mcr-9*-carrying plasmid persisted in *S. nevei* biofilms under our experimental conditions. Using PathogenFinder v.2.1 and VirulenceFinder v.2.0, all the *mcr-9*-positive *S. nevei* were predicted to be potential human pathogens and carried virulence genes. *Serratia* spp. include opportunistic pathogens that can cause different infections (23). Notably, *S. nevei* is closely related to the opportunistic enteric pathogen, *S. marcescens*, which can also cause gastrointestinal infections (24). The SNP distances between the *mcr-9*-positive strains, SFG-21 (in shrimp samples from Indonesia), SFG-24 (Indonesia), SFG-35 (Thailand), and SFG-37 (Thailand) (Table 1), ranged between 23 and 37, while the distance between these strains and SFG-36 (Indonesia) ranged between 16,407 and 16,416. Given that a relatively low number of SNPs (in the tens) is commonly indicative of clonality, it appears that SFG-21, SFG-24, SFG-35, and SFG-37 are more genetically related and potentially clonal.

Here, we identified new sources of *mcr* in the USA, namely imported scallops and shrimps. The *mcr*-carrying bacteria were isolated from packaged and ready-to-eat/cook food samples, indicating that (i) the contamination occurred pre- or during packaging at the country of origin, and (ii) consumers might be directly exposed to these bacteria. Notably, *S. nevei*, carrying *mcr-9* on IncHI2 plasmids, was also detected in sewage samples in Georgia, USA (11). Taken together, this study demonstrates the potential role of food imports in introducing important ARGs, including *mcr*, to the USA. Also, it highlights the need for expanding epidemiological investigations to include intrinsically colistin-resistant bacteria to better understand their role in the dissemination of *mcr*. Lastly, it is important to investigate whether the introduction of transmissible *mcr* via imported foods will result in establishing these genes in the USA.

## Data Availability

The WGS files were deposited in NCBI GenBank under the project “Antimicrobial resistance in local and imported seafood” withaccession no. PRJNA887443. The sequences of the *mcr-9*-positive *Serratia nevei* are available under accession numbers JAROCT000000000, JAROCU000000000, JAROCV000000000, JAROCW000000000, and JAROCX000000000, while the sequence of *mcr-3*-positive *A. salmonicida* was deposited under JAROCS000000000.
